# An Unusual Cause of Thigh Swelling: Extramedullary Myeloid Tumor

**DOI:** 10.4274/tjh.2013.0280

**Published:** 2014-06-10

**Authors:** Memiş Hilmi Atay, Engin Kelkitli, Piltan Büyükkaya, Kubilay Ekiz, Levent Yıldız, Mehmet Turgut

**Affiliations:** 1 Van Training and Research Hospital, Department of Internal Medicine, Division of Hematology, Van, Turkey; 2 Erzurum Training and Research Hospital, Department of Internal Medicine, Division of Hematology, Erzurum, Turkey; 3 Ondokuz Mayıs University Faculty of Medicine, Department of Internal Medicine, Division of Hematology, Samsun, Turkey; 4 Ondokuz Mayıs University Faculty of Medicine, Department of Internal Medicine, Samsun, Turkey; 5 Ondokuz Mayıs University Faculty of Medicine, Department of Pathology, Samsun, TurkeY

**Keywords:** Acute myeloblastic leukemia, Granulocytes, Acute leukemia, Hemophagocytic lymphohistiocytosis

## TO THE EDITOR

Extramedullary myeloid tumor (EMMT) is a rare neoplasm of immature myeloid cells that arises at an extramedullary site [1]. The most common sites of EMMT are the bone, lymph nodes, skin, and soft tissue [2]. EMMT rarely infiltrates the lower extremities. 

**Figure 1:**
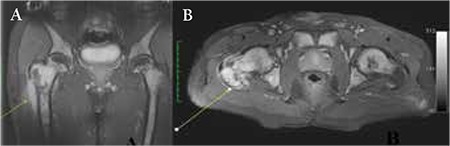
A) Metaphysodiaphyseal paracortical heterogeneous enhancement of the proximal femur in the post-contrast T1-weighted coronal image. B) Paracortical minimal signal increase is seen in the proximal femoral metaphysodiaphyseal part in coronal T2-weighted fat-suppressed image.

**Figure 2:**
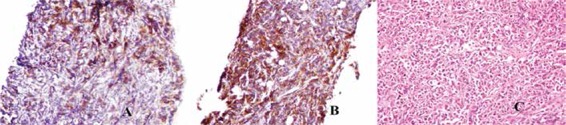
A) Selected cytoplasm, large hyperchromatic nuclei, and tumor infiltration of cells showing marked pleomorphism (H&E, 40x). B) Diffuse strong membranous/cytoplasmic staining for CD34 in tumor cells and associated vascular structures (H&E, 40x). C) Membranous/cytoplasmic staining strong in some places and moderate in some places in tumor cells (CD117, 40x).

A 47-year-old male patient was admitted to the orthopedics clinic because of swelling and pain in the right thigh for 1 month. His past medical history was unremarkable. Physical examination indicated a 10 cm-long solid soft tissue lesion in the anterior and lateral parts of the right thigh. Complete blood count results were as follows: white blood cell count of 15.1x109/L, hemoglobin level of 11.9 g/dL, and platelet count of 94x10^9^/L. Magnetic resonance imaging (MRI) of the right thigh demonstrated a heterogeneous mass extending towards the distal part in the anterolateral section of the right femoral neck, completely involving the vastus lateralis and intermedius muscles (Figure 1). The patient underwent Tru-Cut biopsy of the right thigh. Pathology of the Tru-Cut biopsy showed large blastic cells infiltrating the soft tissue with hyperchromatic nuclei stained positively with CD117, CD34, and myeloperoxidase (Figure 2). After the Tru-Cut biopsy, blood count results were: white blood cells, 21.5x10^9^/L, hemoglobin, 11.5 g/dL, and platelets, 74x10^9^/L. Bone marrow aspiration showed 60% blasts, which were intensely positive for myeloperoxidase. Flow cytometry performed on the bone marrow revealed a blast population that expressed CD34, CD117, CD33, CD15, CD13, CD19, and HLA-DR. As a result of cytogenetic testing, a new complex karyotype related to chromosomes 8, 10, and 21 and trisomy 8 were detected. The patient was started on an acute myeloid leukemia (AML) induction chemotherapy regimen consisting of idarubicin (12 mg/m^2^, daily for 3 days) and cytosine arabinoside (ara-C; 200 mg/m2 continuous infusion for 7 days). After 4 weeks, the control bone marrow aspiration was completely normal. The lesion had also disappeared completely in the control MRI of the thigh. The patient was administered a high-maintenance dose of ara-C at 3 g/m2 for 6 days for consolidation. Treatment is ongoing.

EMMTs are composed of myeloid blasts. They can easily be confused with lymphomas or soft tissue sarcomas [3,4]. EMMTs may accompany AML in 35% of patients at presentation, 38% of patients following diagnosis of AML, and 27% of patients without diagnosis of AML [5]. Cytogenetic abnormalities like translocation (8;22) or inversion 16 and 11q23 were reported in EMMT [6]. An optimal treatment approach does not exist due to the lack of randomized studies. Intensive chemotherapy regimens containing idarubicin plus ara-C are usually administered in the treatment of EMMT. According to the risk factors (age; molecular and cytogenetic study results), allogenic stem cell transplantation may also be considered. In the case of residual infiltration as shown by imaging, radiotherapy should be considered [7].

Consequently, EMMT might be taken into consideration in the differential diagnosis of venous thromboembolism and soft tissue malignancies in the case of swollen thighs.

## CONFLICT OF INTEREST STATEMENT

The authors of this paper have no conflicts of interest, including specific financial interests, relationships, and/ or affiliations relevant to the subject matter or materials included.
